# Development of novel cellular histone-binding and chromatin-displacement assays for bromodomain drug discovery

**DOI:** 10.1186/s13072-015-0026-4

**Published:** 2015-09-21

**Authors:** Yanai Zhan, Maria Kost-Alimova, Xi Shi, Elisabetta Leo, Jennifer P. Bardenhagen, Hannah E. Shepard, Srikanth Appikonda, Bhavatarini Vangamudi, Shuping Zhao, Trang N. Tieu, Shiming Jiang, Timothy P. Heffernan, Joseph R. Marszalek, Carlo Toniatti, Giulio Draetta, Jessica Tyler, Michelle Barton, Philip Jones, Wylie S. Palmer, Mary K. Geck Do, Jannik N. Andersen

**Affiliations:** Institute for Applied Cancer Science, The University of Texas MD Anderson Cancer Center, Houston, TX 77230 USA; Department of Epigenetics and Molecular Carcinogenesis, The University of Texas MD Anderson Cancer Center, Houston, TX 77230 USA; XTuit Pharmaceuticals, 700 Main Street, Cambridge, MA 02139 USA

**Keywords:** Bromodomain–histone-binding assays, Chromatin drug-target displacement, AlphaLISA, AlphaScreen, In situ cell extraction, TRIM24, Bromodomain inhibitor, IACS-9571, IACS-6558

## Abstract

**Background:**

Proteins that ‘read’ the histone code are central elements in epigenetic control and bromodomains, which bind acetyl-lysine motifs, are increasingly recognized as potential mediators of disease states. Notably, the first BET bromodomain-based therapies have entered clinical trials and there is a broad interest in dissecting the therapeutic relevance of other bromodomain-containing proteins in human disease. Typically, drug development is facilitated and expedited by high-throughput screening, where assays need to be sensitive, robust, cost-effective and scalable. However, for bromodomains, which lack catalytic activity that otherwise can be monitored (using classical enzymology), the development of cell-based, drug-target engagement assays has been challenging. Consequently, cell biochemical assays have lagged behind compared to other protein families (e.g., histone deacetylases and methyltransferases).

**Results:**

Here, we present a suite of novel chromatin and histone-binding assays using AlphaLISA, in situ cell extraction and fluorescence-based, high-content imaging. First, using TRIM24 as an example, the homogenous, bead-based AlphaScreen technology was modified from a biochemical peptide-competition assay to measure binding of the TRIM24 bromodomain to endogenous histone H3 in cells (AlphaLISA). Second, a target agnostic, high-throughput imaging platform was developed to quantify the ability of chemical probes to dissociate endogenous proteins from chromatin/nuclear structures. While overall nuclear morphology is maintained, the procedure extracts soluble, non-chromatin-bound proteins from cells with drug-target displacement visualized by immunofluorescence (IF) or microscopy of fluorescent proteins. Pharmacological evaluation of these assays cross-validated their utility, sensitivity and robustness. Finally, using genetic and pharmacological approaches, we dissect domain contribution of TRIM24, BRD4, ATAD2 and SMARCA2 to chromatin binding illustrating the versatility/utility of the in situ cell extraction platform.

**Conclusions:**

In summary, we have developed two novel complementary and cell-based drug-target engagement assays, expanding the repertoire of pharmacodynamic assays for bromodomain tool compound development. These assays have been validated through a successful TRIM24 bromodomain inhibitor program, where a micromolar lead molecule (IACS-6558) was optimized using cell-based assays to yield the first single-digit nanomolar TRIM24 inhibitor (IACS-9571). Altogether, the assay platforms described herein are poised to accelerate the discovery and development of novel chemical probes to deliver on the promise of epigenetic-based therapies.

**Electronic supplementary material:**

The online version of this article (doi:10.1186/s13072-015-0026-4) contains supplementary material, which is available to authorized users.

## Background

Histone acetylation is a well-characterized modification that is regulated by opposing activities of histone acetyltransferases (HATs) and histone deacetylases (HDACs). The histone code hypothesis, originally proposed a decade ago [1], suggests that specific combinations of histone post-translational modifications (PTMs) constitute an epigenetic marking system that determines distinct transcriptional and functional outputs of eukaryotic genomes. These epigenetic marks can be ‘read’ by proteins that specifically bind to single or combined histone PTMs [2].

In humans, recognition of acetyl lysine is accomplished by a conserved family of 61 bromodomains encoded by 42 human genes [3]. Many bromodomain-containing proteins have additional regulatory and protein–protein interaction domains, which contribute in a combinatorial manner to highly specialized functions as transcriptional regulators, chromatin remodelers, splicing factors, scaffolding proteins and signal transducers. For example, some bromodomains are flanked by catalytic domains with ATPase, methyltransferase or acetyltransferase enzymatic activity but, in most cases, the functional contribution of bromodomains to the biological activity of multi-domain proteins remains unknown [3]. Nevertheless, with the first BET bromodomain-based therapies in clinical trials showing early signs of efficacy, bromodomains are increasingly recognized as mediators of a wide range of disease states and, as such, offer attractive candidate therapeutic targets [4].

A key step in the drug discovery process is optimization of chemical probes using relevant biochemical and cellular assays. Typically, the identification of small-molecule inhibitors is facilitated and expedited by high-throughput screening (HTS), where assays need to be sensitive, robust, cost-effective and scalable [5]. For bromodomains, which lack catalytic activity that otherwise can be monitored (i.e., using classical enzymology), the development of cell-based, drug-target engagement assays to support probe development has been challenging. Proximity-based, resonance energy transfer methods (where excited-state energy is transferred from one fluorophore to another) have been widely used to detect protein–protein interactions in living cells [6], and recently, a bioluminescence-based assay (nanoBRET) was developed using cells expressing luciferase-tagged BRD4 and histone H3.3-HT fusion proteins [7]. Likewise, a time-resolved fluorescence resonance energy (TR-FRET) assay has been developed to quantify the interaction of transfected BRD4 bromodomain with chemical inhibitors inside cells based on ligand-induced protein stabilization [8]. Historically, however, Fluorescence Recovery After Photobleaching (FRAP), which requires confocal laser scanning microscopy, has been the most widely used assay to determine differences in diffusion rates of GFP-tagged bromodomains in the presence or absence of small-molecule inhibitors [9]. As described, the cellular FRAP assay can detect whether a compound can modulate chromatin binding (i.e., the diffusion rate) of transfected GFP-tagged bromodomains, but the FRAP assay lacks sufficient sensitivity or throughput to rank-order inhibitor potency to drive aggressive lead optimization programs. This is in contrast to the histone methyltransferase field where multiple homogenous, cell-based assays are commercially available using AlphaLISA and LanthaScreen technologies, as exemplified by Histone tri-methylation (H3K27Me3) kits for the development of EZH2 inhibitors [10]. Hence, what is needed for bromodomain drug discovery is a convenient, cell-based method, suitable for HTS, to measure displacement of bromodomains from histones or chromatin.

Here, we describe three bromodomain-binding assays, using TRIM24 as an example: one in vitro assay that measures histone peptide (H3K23Ac) binding to TRIM24 and two novel cell-based assays that capture TRIM24 binding to either endogenous histone H3 or chromatin/nuclear structures in cells. The two cellular assays use orthogonal detection methods: amplified luminescent proximity bead-based technology (AlphaLISA) and fluorescent high-content imaging, respectively. To our knowledge, these are the first quantitative, high-throughput methods for the direct visualization of bromodomain–histone binding and inhibitor-mediated disruption of binding in cells, miniaturized to 384-well plate format with excellent plate statistics and assay performance. The in situ cell extraction protocol, coupled with high-content IF imaging, is target agnostic and can in principle be used to quantify the displacement of any protein of interest from chromatin in any target cell, thereby expanding the repertoire of epigenetic HTS assays for chemical probe discovery.

## Results and discussion

### Considerations when identifying peptide ligands for bromodomain assay development

**Fig. 1:**
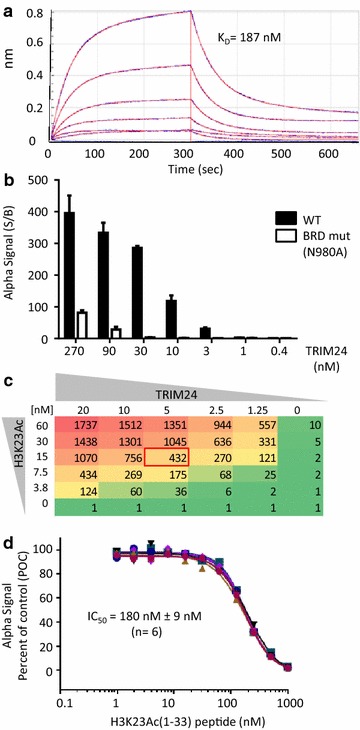
Characterization of the H3K23Ac peptide ligand for the biochemical TRIM24 AlphaScreen assay development. **a** Binding affinity (*K*_*D*_) of the His-TRIM24-PB/H3K23Ac-peptide interaction calculated from kinetic fitting of the association and dissociation profiles measured by biolayer interferometry. **b** AlphaScreen signal to background (S/B) for His-TRIM24-PB binding to the biotinylated-H3K23Ac peptide (1 µM) using the indicated amounts of wild-type (WT) and bromodomain mutant (N980A) protein. **c** AlphaScreen assay optimization using a matrix of TRIM24 protein and peptide (i.e., twofold dilution series starting from 20 to 60 nM, respectively). The Alpha Signal (S/B) is the average of 2 independent 384-well plates highlighting conditions (*red box*) selected for the final assay protocol (Additional file 1: Table S1). **d** AlphaScreen peptide competition and dose–response validation. Non-biotinylated H3K23Ac peptide was titrated into a solution of His-TRIM24-PB (5 nM) and biotinylated-H3K23Ac peptide (15 nM) with IC_50_ curves shown from 6 independent plates

Prior to embarking on the development of cell-based bromodomain assays, we first established biochemical assays to identify small-molecule inhibitors of recombinant purified bromodomains. The identification of sufficiently tight-binding peptides is usually a key challenge when developing in vitro bromodomain-binding assays, as illustrated by our work on ATAD2 [11], where a combination of crystallography, empirical screening of peptides and mutagenesis was required to identify an active-site ligand (H4K5Ac) suitable for small-molecule HTS (Additional file 1: Figure S1). Dissociation constants between bromodomains and synthetic peptides are typically in the micromolar or millimolar range [12]; however, since most bromodomains are present in large multi-domain proteins, it is appreciated that bromodomains often act in concert with other regulatory domains. For example, the adjoining plant homeodomain (PHD) and bromodomain in TRIM24 function as a single unit in combinatorial recognition of unmodified H3K4 and H3K23Ac within the same histone tail [13]. Notably, this domain architecture confers enhanced avidity towards the corresponding histone H3 peptide (*K*_D_ of 96 nM) as measured by ITC [13]. Here, we expressed and purified recombinant PHD-bromodomain (His-TRIM24), which yielded a *K*_D_ of 187 nM (Fig. 1a) for a biotinylated H3K23Ac peptide (N-terminal residues 1–33 of histone H3) using biolayer interferometry. Hence, we conclude that the biotin tag on the synthetic H3K23Ac peptide minimally interferes with TRIM24 bromodomain binding, highlighting the utility of this labeled peptide for development of a bromodomain/peptide-displacement assay.

### Development of a TRIM24/peptide-displacement AlphaScreen assay

To explore the development of an HTS-friendly and homogenous ligand binding assay, purified recombinant wild-type (WT) and mutant (N980A) His-TRIM24 were incubated with the biotinylated H3K23Ac peptide, followed by sequential addition of Streptavidin-coated donor beads and Ni–NTA acceptor beads. Clearly, similar to AlphaScreen assays for BRD4 and ATAD2 [11, 12], formation of the TRIM24/H3-peptide complex brought the donor and acceptor beads into proximity of each other with laser irradiation of the donor beads (680 nm) resulting in chemiluminescent emission (570 nm) of nearby acceptor beads (Fig. 1b). With the H3K23Ac peptide in excess (1 µM), the AlphaSignal for TRIM24 WT was dosage proportional up to 30 nM affording a signal to background (S/B) of >250, with no detectable signal over background for the bromodomain-binding-deficient mutant (N980A) harboring a classical mutation of a conserved asparagine required for coordination and binding of acetyl lysine [3]. After automation of liquid handling steps, the final assay conditions selected were 5 nM TRIM24 and 15 nM peptide, which provided an assay window with a S/B ratio of 432 (Fig. 1c; Additional file 1: Table S1). Finally, given the absence of small-molecule TRIM24 inhibitors at the time of assay development, we titrated a non-biotinylated H3K23Ac peptide into the assay and confirmed dose-dependent competition against the biotinylated sister peptide measuring an IC_50_ of 180 nM (Fig. 1d). Collectively, these experiments validated the TRIM24 AlphaScreen assay for chemical library screening [11, 14] and we embarked on developing a suite of cellular assays to follow-up on the HTS campaign.

### Characterization of TRIM24 bromodomain binding to histone H3 in cells

**Fig. 2:**
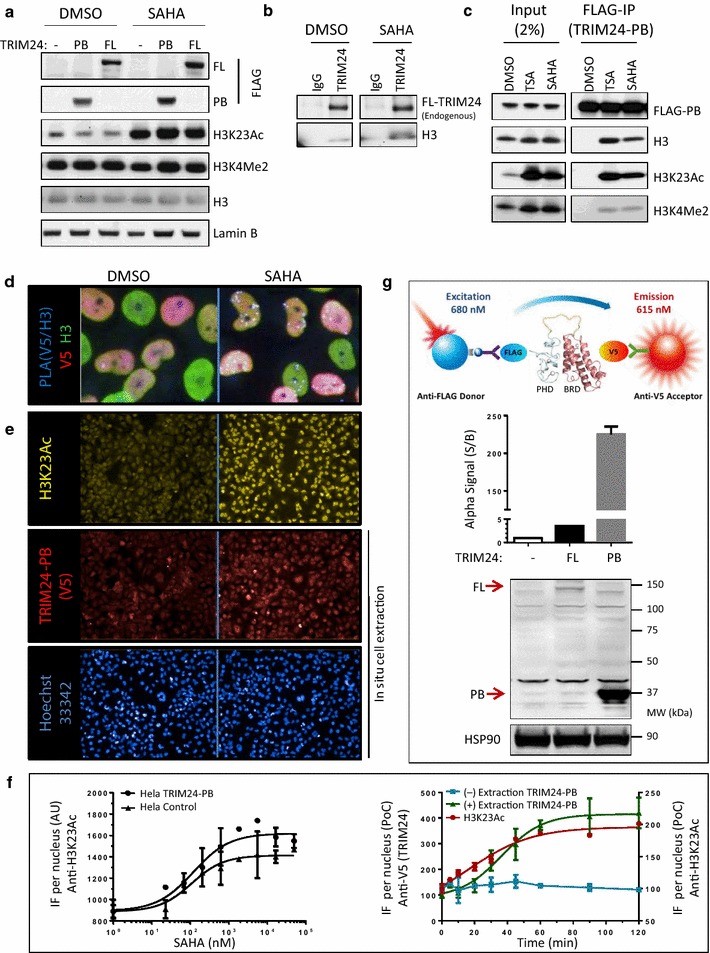
Characterization of engineered HeLa cells expressing TRIM24 full-length or isolated PHD-bromodomain. **a** Western blot analysis of stable HeLa cell lines expressing either vector control (−) or dual-tagged (FLAG-TRIM24-V5) full-length (FL) or isolated PHD-bromodomain (PB). Lysate from cells treated with Suberoylanilide Hydroxamic Acid (SAHA) for 2 h (2 µM) or vehicle control (DMSO) were probed with the indicated antibodies confirming SAHA-induced acetylation of H3K23. **b** Immunoprecipitation of endogenous full-length TRIM24 (using anti-TRIM24 antibody) under non-treated (DMSO) and SAHA-treated (2 µM, 2 h) conditions. **c** Immunoprecipitation of the TRIM24 PHD-bromodomain (using anti-FLAG beads) and looking at co-immunoprecipitated histone H3 in TRIM24-PB cells non-treated or treated (2 µM, 2 h) with either Trichostatin A (TSA) or (SAHA). **d** Proximity ligation assay (PLA) of TRIM24-PB cells using the goat anti-V5 (i.e., TRIM24-PB) and mouse anti-histone H3 antibody pair, showing an increase in co-localization of TRIM24 with histone H3 in response to SAHA treatment (2 µM, 2 h). The increase in proximity of the two proteins (*number of* *blue PLA dots*) is quantified in Additional file 1: Figure S2. **e** Representative IF images of TRIM24-PB cells following SAHA treatment (2 µM, 2 h) showing acetylation of histone H3K23 (*yellow*) and increased chromatin binding of TRIM24-PB (*red*) with Hoechst nuclear staining (*blue*) serving as a control for the in situ cell extraction procedure. **f** SAHA dose–response and time-course studies. *Left Graph* IF quantification (Arbitrary Units) of H3K23Ac in Hela control and TRIM24-PB cells upon SAHA treatment (2 h). *Right Graph* IF quantification (average IF signal per nucleus for TRIM24-PB and H3K23Ac as percent of the non-SAHA-treated control (PoC)) under in situ cell extraction (+) and non-extraction (−) conditions. **g** Schematic diagram for measuring the dual-tagged TRIM24 protein in cell lysates (*Top*) showing AlphaLISA results for HeLa cells (Parental, TRIM24-FL and TRIM24-PB) using anti-FLAG donor and anti-V5 acceptor beads (*Middle*) compared to immunoblotting (*Bottom*)

To develop cellular TRIM24 binding assays for drug-target engagement studies, we generated stable HeLa cell lines expressing full-length TRIM24 (TRIM24-FL) or isolated PHD-bromodomain (TRIM24-PB) as shown by Western blotting (Fig. 2a). Both constructs were dual epitope tagged with FLAG at the N-terminus and V5 at the C-terminus. In response to HDAC inhibitor treatment with Trichostatin A (TSA) or Suberoylanilide Hydroxamic Acid (SAHA), the cellular histone H3K23Ac mark that TRIM24 bromodomain binds becomes hyperacetylated (Fig. 2a, b). Importantly, anti-FLAG immunoprecipitation (IP) of TRIM24 was able to co-immunoprecipitate (Co-IP) histone H3 from cells. However, in contrast to IP of endogenous full-length TRIM24 (Fig. 2b), the isolated PHD-bromodomain (TRIM24-PB) formed a detectable immune complex with endogenous histone H3 only when cells were pretreated with HDAC inhibitor to increase overall histone acetylation levels (Fig. 2c). Notably, compared to the input lysate, the immunoprecipitated histone H3 showed minimal H3K4 methylation levels relative to H3K23 acetylation (Fig. 2c), consistent with the specific TRIM24-PB recognition motif defined by X-ray crystallography (i.e., H3K4 methylation of the H3K23Ac(1–33) peptide reduces its binding affinity to the TRIM24 PHD-bromodomain through steric hindrance) [13]. Interestingly, HDAC inhibitors are known to induce H3K4 methylation [15, 16], and since this mark is tightly associated with promoters of active genes [17], our Co-IP studies suggest that TRIM24 is not recruited to this subset of transcriptional active promoters [18].

To further characterize recruitment of TRIM24-PB to acetylated histone H3 in intact cells, we next applied a proximity ligation assay (PLA) [19] to visualize the SAHA-induced binding using a pair of anti-V5 and anti-histone H3 antibodies (Fig. 2d; Additional file 1: Figure S2). Indeed, SAHA treatment (2 h, 5 µM) led to a greater than threefold increase in the TRIM24/H3 PLA signal (Fig. 2d—blue dots), whereas mean nuclei levels of TRIM24 did not change, as measured by Alexa-conjugated secondary antibodies (Additional file 1: Figure S2c). Altogether, the TRIM24 co-IP and PLA co-localization studies suggested that the engineered HeLa cells are a suitable model system for development of cellular bromodomain–histone-binding assays to support small-molecule drug discovery.

### Development of cellular TRIM24-histone AlphaLISA binding assay: selection of cell line

To identify optimal conditions for TRIM24 binding to endogenous histone H3 in cells, we next conducted SAHA time– and dose–response studies using anti-H3K23Ac immunostaining (Fig. 2e—yellow). We observed that treatment with 3 µM SAHA for 2 h is sufficient to drive maximal H3K23 acetylation in both HeLa control and TRIM24-PB expressing cells (Fig. 2f—left graph). Prior to paraformaldehyde fixation, we introduced an in situ cell extraction step, similar to methods previously applied to yeast cells [20, 21], to remove soluble, non-chromatin-bound proteins. Under these conditions, we observed that TRIM24-PB became resistant to detergent extraction, indicating that the PHD-Bromodomain bound more tightly to chromatin and/or related nuclear structures with SAHA treatment (Fig. 2e—red) while total cell number (Hoechst) remained unchanged (Fig. 2e—blue). Notably, the chromatin binding of TRIM24-PB occurred concomitantly with SAHA-induced acetylation of H3K23Ac (Fig. 2f—right graph), consistent with a direct TRIM24 protein–histone interaction.

Finally, to explore the feasibility of a homogenous, proximity-based AlphaLISA assay, we next incubated HeLa cell lysates (parental, TRIM24-FL or TRIM24-PB) with anti-FLAG and anti-V5 antibodies conjugated to acceptor and donor beads, respectively, as detection reagents. As illustrated in the cartoon (Fig. 2g—top), this allowed us to evaluate the intensity (and specificity) of the AlphaLISA signal, leveraging the dual-tagged (FLAG and V5) TRIM24 protein as a best-case scenario for assay sensitivity. Clearly, the AlphaLISA was sensitive enough to detect expression of full-length, tagged TRIM24, which was overexpressed two- to threefold above endogenous levels (Fig. 2g—middle). In comparison, the HeLa cell line expressing TRIM24-PB produced an AlphaLISA S/B of 220, consistent with the higher expression level of the smaller PHD-bromodomain construct evident from the anti-TRIM24 Western blot (Fig. 2g—bottom). Robust detection of dual-tagged TRIM24-PB (S/B > 200) in lysates obtainable from a single well (i.e., 10,000 cells in a 96-well plate format) was encouraging as it implies, at least theoretically, the ability to detect TRIM24-PB in complex with endogenous histone H3 in a high-throughput format. Here, motivated by a desire to have a high AlphaLISA signal for small-molecule library screening, we hence proceeded with the HeLa TRIM24-PB cell line for HTS assay development.

### Development of a cellular TRIM24-histone AlphaLISA assay: optimization of cell lysis and histone extraction buffers to preserve bromodomain/histone interactions

**Fig. 3:**
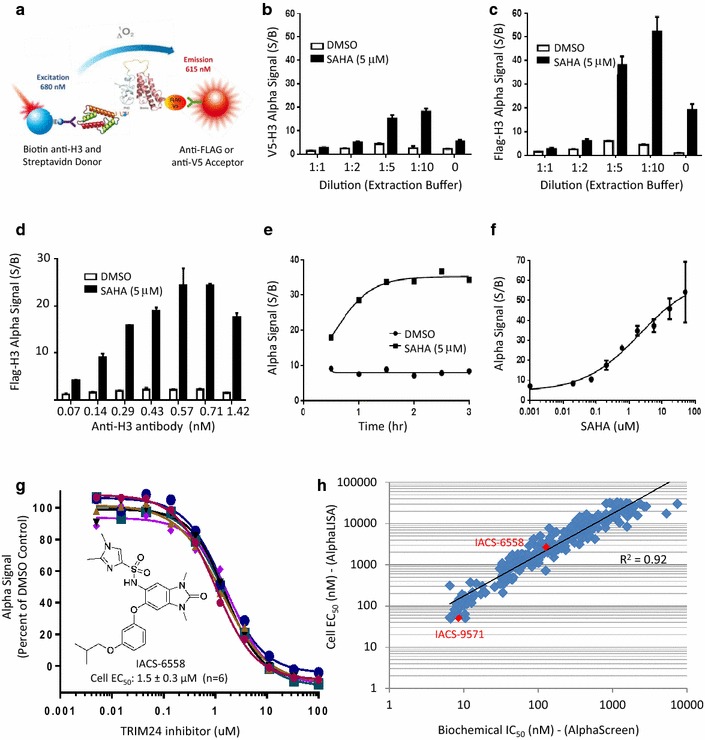
Cellular AlphaLISA assay development to detect bromodomain–histone H3 interactions in HTS format. **a** Schematic diagram for quantifying the association of TRIM24 and histone H3 in cells using AlphaLISA. Streptavidin-coated donor beads capture the immune complex between the biotinylated anti-H3 antibody and histone H3. Acceptor beads (directly conjugated to either anti-FLAG or anti-V5 antibodies) captures TRIM24. If a test agent displaces TRIM24 from histone H3, it will lead to decreased Alpha Signal. **b**, **c** Stringency of histone extraction evaluated for HeLa TRIM24-PB cells pretreated with either DMSO or SAHA (5 µM, 2 h) using the indicated AlphaLISA acceptor beads (V5 or FLAG) and a dilution series of the histone extraction buffer. **d** Titration of the anti-histone H3 antibody with FLAG-acceptor beads (5 mg/mL). **e** SAHA time-course (5 µM) and **f** dose–response (2 h) studies for TRIM24-PB HeLa cells plated in 384-well plates and subjected to the AlphaLISA protocol (Additional file 1: Table S2). **g** Dose–response curves for cells treated with IACS-6558, the depicted small-molecule TRIM24 bromodomain inhibitor (TRIM24i). Inhibitor affinity (EC_50_) is shown as percent binding relative to control (DMSO) from 6 independent experiments. **h** Correlation plot between biochemical AlphaScreen (IC_50_) and cellular AlphaLISA (EC_50_) values for 273 compounds evaluated during our TRIM24 bromodomain drug discovery program [14]

Using the depicted AlphaLISA reagents (Fig. 3a), we next explored whether we could detect a TRIM24/histone H3 complex in cell lysates. Specifically, either V5- or FLAG-conjugated acceptor beads were paired with a biotinylated anti-histone H3 antibody, followed by addition of Streptavidin-coated donor beads and signal acquisition (Fig. 3b, c). Not surprisingly, the default (i.e., manufacturer recommended) cell lysis and histone extraction protocol for the anti-H3 AlphaLISA antibody [10], which have been optimized to capture histone H3 and measure levels of H3K27 tri-methylation, which is a covalent modification, were too harsh to maintain specific bromodomain–histone protein–protein interaction. Hence, we explored a range of different lysis conditions by incubating V5/H3 (Fig. 3b) and FLAG/H3 (Fig. 3c) antibody pairs under less stringent conditions, initially diluting the Histone Extraction Buffer up to tenfold in water (1:1, 1:2, 1:5 and 1:10). Using the lysis buffer defined in Additional file 1: Table S2, a significant immune complex between TRIM24-PB and endogenous histone H3 emerged when the Histone Extraction Buffer was diluted 1:5 or more (Fig. 3b, c). Specifically, a 50-fold assay window (S/B) was found for FLAG-acceptor and Streptavidin-donor beads, using a 1:10 dilution of the histone extraction buffer (Fig. 3c), compared to 14-fold (S/B) for V5-acceptor and Streptavidin-donor beads (Fig. 3b). Replacing the Histone Extraction Buffer entirely with water reduced the S/B from 50 to 20 for FLAG-acceptor beads (Fig. 3c). Of note, we also explored enzymatic means of digesting chromatin (i.e., using micrococcal nucleases) and varied the ionic strength and pH of the lysis buffer to see whether these conditions could further help expose the epitope recognized by the biotinylated H3 antibody; however, none of these efforts further improved the S/B to reach levels above 50. Therefore, we proceeded with assay development as described below.

### Optimization (384-well plate) and pharmacological validation of the AlphaLISA assay

For the final AlphaLISA assay optimization, we titrated the amount of biotinylated H3-antibody (keeping FLAG-acceptor and Streptavidin-donor beads constant) and observed the typical ‘hook’ effect, a well-understood phenomenon for saturable bimolecular detection systems [22], at 0.57 nM histone H3 antibody (Fig. 3d). Hence, we selected 0.5 nM as the optimal H3 antibody concentration and repeated the SAHA dose- and time-course studies (Fig. 2e), now detecting the formation of the TRIM24/histone complex directly in cells processed in 384-well tissue culture plates (Fig. 3e, f). The kinetics of the TRIM24/histone-H3 complex formation as measured by AlphaLISA was consistent with that obtained by IF (Fig. 2e), validating the final protocol for our small-molecule drug discovery program (Additional file 1: Table S2).

Titration curves for IACS-6558, a TRIM24 bromodomain inhibitor (TRIM24i) identified during our structure-guided drug discovery program [14] are highlighted to document the sensitivity and reproducibility of the AlphaLISA assay affording an IC_50_ of 1.5 ± 0.3 µM (Fig. 3g). The assay performance and plate statistics (sampled from 26 plates; 384-well format) are clearly acceptable for a cell-based assay (S/B of 30; S/N of 11; *Z*′ of 0.67) [23]. Moreover, as part of the lead optimization process, we observed a strong correlation (*R*^2^ = 0.92) between affinities for chemical matter tested in both the biochemical TRIM24/histone-peptide-displacement assay (AlphaScreen) and the cellular TRIM24/histone-binding assay (AlphaLISA) (Fig. 3h). Ultimately, combined with molecular structure-guided insights (i.e., X-ray crystallography), this enabled us to develop drug-like probes with minimal cell-shift and single-digit nanomolar activity, as exemplified by IACS-9571 [14].

### In situ cell extraction as an orthogonal approach to study bromodomain/chromatin binding

**Fig. 4:**
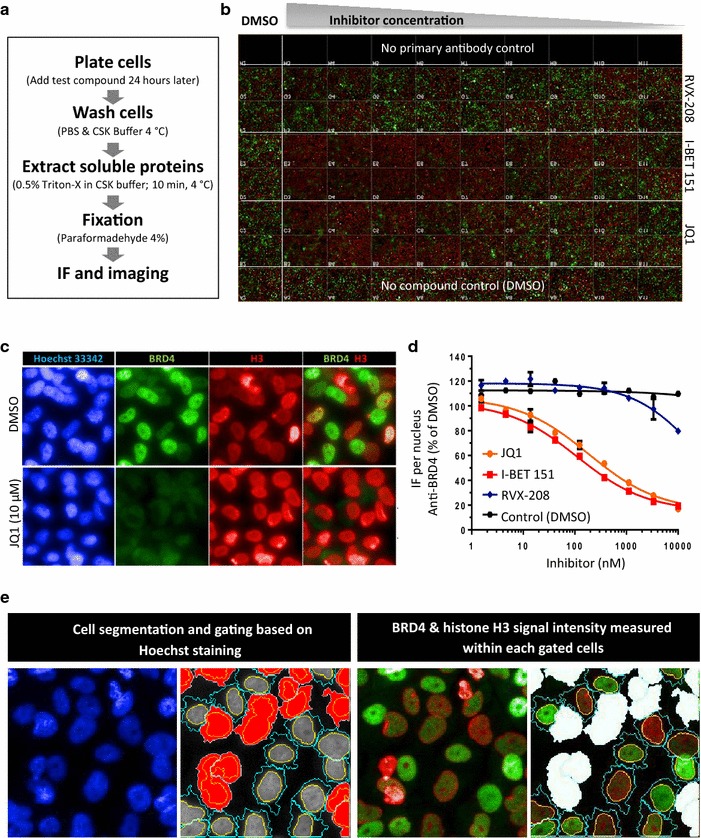
Pharmacological proof-of-concept for the in situ cell extraction methodology using reference BET bromodomain inhibitors. **a** Outline of the in situ cell extraction procedure. **b** 96-well plate view for BET bromodomain inhibitor treatment of HeLa cells expressing FLAG-tagged full-length BRD4. Cells were subjected to in situ cell extraction and two-color IF staining for TRIM24 (*green*) and histone H3 (*red*). The plate layout shows serial dilution of 3 BET inhibitors (JQ1, RVX-280 and I-BET) including wells with DMSO and secondary antibody controls. **c** Magnified IF images show decrease of BRD4 staining upon JQ-1 treatment (10 µM), while histone H3 (*red*) and Hoechst staining remains unchanged. **d** Quantification of drug dose responses based on IF image analysis. **e** Image analysis sequence using Harmony software. *Left panel* cell segmentation and gating strategy based on Hoechst (*blue*) staining eliminating cells of inappropriate morphology (*red on the second image*). *Right panel* the same cells with co-staining for BRD4 (*green*) and H3 (*red*). The mean intensity of the BRD4 signal was measured per nucleus and the average nucleus intensity was calculated from about 1000 gated cells (i.e., excluding the masked white cells on the fourth image)

Chromatin binding of proteins has been studied using various biochemical- and fluorescence-based imaging techniques in both fixed and live cells. Specifically, in situ cell extraction techniques, which remove proteins not bound to nuclear structures, have been used to study chromatin binding of individual proteins involved in chromatin remodeling, cell cycle and DNA repair [24]. Our observation that TRIM24 protein levels in the non-soluble, nuclear fraction of cells increased concomitantly with SAHA-induced acetylation (Fig. 3d) suggested that this simple detergent extraction and fixation protocol (Fig. 4a) could be developed into an orthogonal and target agnostic chromatin-binding assay. In principle, such assay would be universally applicable across the bromodomain protein family and potentially other chromatin-associated drug targets.

To this end, we first titrated three reference BET-family bromodomain inhibitors (JQ1 [25], I-BET 151 [26, 27] and RVX-208 [28] using HeLa cells expressing FLAG-tagged BRD4 cultured in 96-well plates (Fig. 4b). Strikingly, using two-color staining, we found that JQ1 and I-BET 151 potently displaced BRD4 (green) from chromatin, while the IF signal for histone H3 (red) was unaffected by inhibitor treatment (Fig. 4b, c). Moreover, BRD4 was displaced from chromatin within 5 min of inhibitor treatment (Additional file 1: Figure S3), consistent with the rapid displacement measured by FRAP [9]. As such, these data support a direct pharmacological effect on binding, rather than a displacement due to secondary drug-induced transcriptional response.

For quantitative image analysis of the drug response (Fig. 4d), we developed a protocol that relied on cell segmentation and gating, based on Hoechst staining followed by the measurement of nuclear IF intensity for BRD4 and H3 (Fig. 4e). Our automated, script-based protocol, allowed rapid acquisition of EC_50_ values using only a small fraction (~10 %) of the cells available in a 96-well plate. Hence, we miniaturized the assay to 384-well format further increasing throughput and reducing reagent costs (Additional file 1: Figure S4). Moreover, the in situ cell extraction assay for BRD4 was exquisitely sensitive and appeared to distinguish between even small intrinsic differences in potency between JQ1 and I-BET 151 (Cell IC_50_ of 132 nM and 86 nM, respectively, compared to BromoScan Kd values of 21 and 10 nM obtained in parallel at DiscoveRx for the recombinant purified proteins [29]). The wide dynamic range and sensitivity of the in situ cell extraction assay was further evident from the partial displacement of BRD4 from chromatin by RVX-208, a compound that selectively binds to the second bromodomain of BET proteins [28, 30]. Interestingly, the BRD4 overexpressing cells do not require SAHA pretreatment to measure drug-target displacement, as the FLAG-tagged BRD4 protein is chromatin bound in the basal, non-hyperacetylated state.

### Simplified and HTS-friendly GFP-version of the in situ cell extraction assays

In an HTS setting, we further simplified the in situ cell extraction procedure by replacing the indirect IF step (e.g., anti-FLAG) with cells engineered to express fluorescent moieties (e.g., GFP) fused to the protein target of interest (Additional file 1: Figure S5). In this format, the assay has minimal time and reagent costs associated with HTS operation, since no further liquid handling or processing steps are needed after detergent extraction and fixation. For example, for the TRIM24 inhibitor, dose–response curves using HeLa cells expressing GFP-tagged TRIM24 and the in situ cell extraction procedure yielded EC_50_ values comparable to the AlphaLISA assay, recording 2.3 µM (Additional file 1: Figure S5B) and 1.3 µM (Fig. 3g), respectively. This is particularly noteworthy for two orthogonal assays that monitor either the displacement of TRIM24 from chromatin/nuclear structures or directly measure TRIM24/histone-H3 dissociation in cells. Moreover, we also note that the assay is robust to the duration of the in situ cell extraction step (4–13 min) affording very similar IC_50_ values (Additional file 1: Figure S6), and we chose a 10-min incubation step for test compounds as the final protocol (Additional file 1: Table S3).

### Pharmacodynamic evaluation of endogenous proteins using in situ cell extraction

**Fig. 5:**
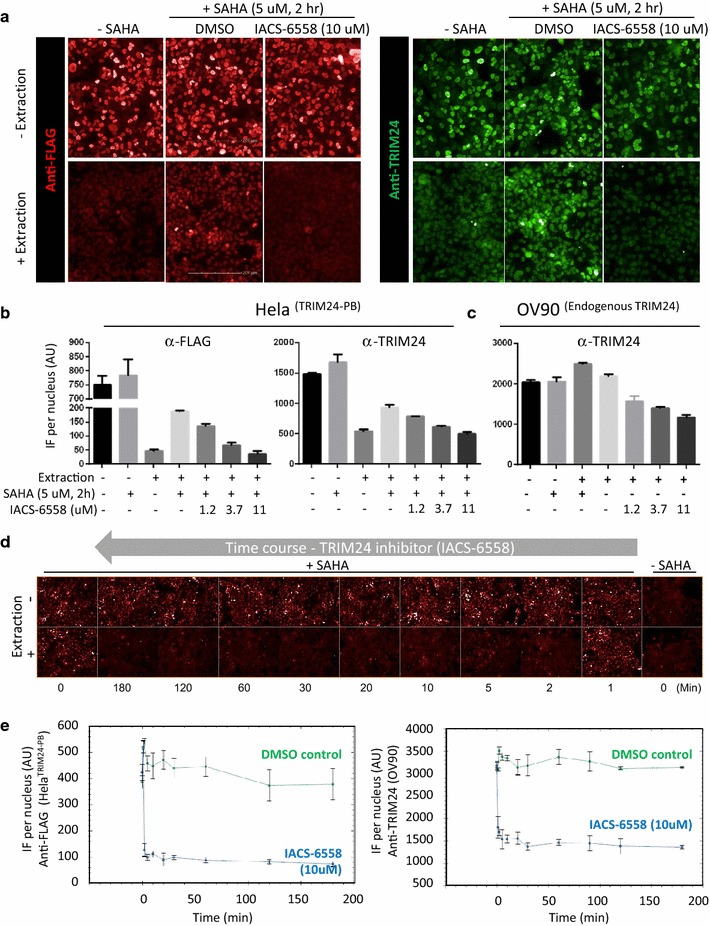
Development of in situ cell extraction to monitor the displacement of endogenous TRIM24 from chromatin in cells. **a** Representative IF images of HeLa TRIM24-PB cells using either anti-FLAG (*red*) or anti-TRIM24 (*green*) antibodies under in situ cell extraction (+) and non-extraction (−) conditions and in the presence or absence of IACS-6558, a TRIM24 inhibitor. **b** Quantification of the fraction of chromatin-bound TRIM24 upon inhibitor treatment (2 h) of the above HeLa TRIM24-PB cells. **c** OV90 cancer cells treated as above using the same anti-TRIM24 antibody to detect endogenous TRIM24 expression and displacement upon TRIM24 inhibitor (IACS-6558) treatment. **d** Representative images (HeLa TRIM24-PB) showing rapid displacement of TRIM24 (*red*) from chromatin in response to TRIM24 inhibitor (IACS-6558) treatment. **e** Quantification of the inhibitor-induced displacement of TRIM24-PB from chromatin in HeLa (*left graph*) and non-transfected OV90 cells looking at endogenous TRIM24 (*right graph*)

In an effort to measure pharmacodynamic changes in the association of endogenous TRIM24 with chromatin in tumor cells, we next validated the utility of an anti-TRIM24 antibody in HeLa TRIM24-PB (Fig. 5a, b) and non-transfected cancer cells (Fig. 5c). In HeLa cells, the anti-TRIM24 antibody provided a smaller assay window for the SAHA-induced TRIM24-PB chromatin binding (twofold), compared to the anti-FLAG antibody (fivefold), but both antibodies recorded similar dose-dependent TRIM24 inhibitor responses reducing the IF signal to baseline (i.e., the level observed without SAHA treatment) (Fig. 5a, b). Together with siRNA knock-down (data not shown), these findings confirmed the specificity and sensitivity of the TRIM24 antibody for IF studies, eliminating the need for an engineered epitope tag. Hence, we qualified the anti-TRIM24 antibody in cancer cells as shown for the ovarian OV90 cell line using a similar treatment matrix (Fig. 5c). We found that the mean IF nuclei intensity did not change in response to SAHA treatment or following in situ cell extraction indicating that endogenous TRIM24 is firmly bound to chromatin under physiological conditions (i.e., present in the non-soluble nuclear fraction of cells). However, the chromatin binding of endogenous TRIM24 was readily reduced in response to pharmacological TRIM24 inhibition (IACS-6558), which clearly is a more relevant PD biomarker assay compared to the displacement of exogenously expressed PhD-bromodomain. Furthermore, the rapid displacement of both endogenous TRIM24 and TRIM24-PB from chromatin, which occurs within 5 min of inhibitor treatment (Fig. 5e), resembles the kinetics observed for displacement of BRD4 by the JQ1 bromodomain inhibitor (Additional file 1: Figure S3). Altogether, these studies support the in situ cell extraction procedure as a potential universal assay platform for studying the pharmacological effects of compounds on chromatin-associated drug targets.

### Cross-validation of cellular and biochemical assays using a panel of TRIM24 inhibitors

**Fig. 6:**
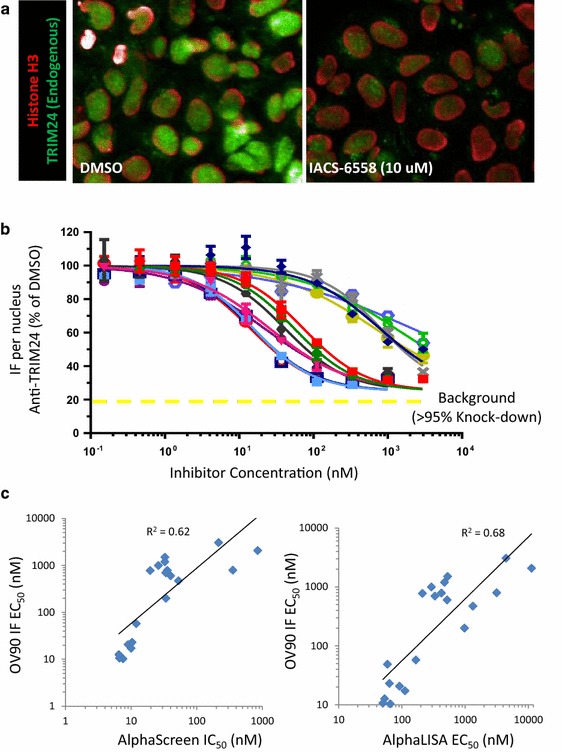
Pharmacological cross-validation of cellular histone and chromatin-binding assays with the TRIM24 biochemical AlphaScreen assay. **a** Merged IF images of two-color staining of OV90 cells following in situ cell extraction showing TRIM24 inhibitor-mediated displacement of endogenous TRIM24 (*green*) from chromatin while leaving histone H3 (*red*) protein levels unchanged (IACS-6558, 10 µM, 2 h). **b** Dose–response curves for a dozen TRIM24 inhibitors (1 h, no SAHA treatment) in OV90 cells using in situ cell extraction and IF detection/quantification. **c** EC_50_ values calculated from the above drug titration studies in OV90 cells (percent inhibition relative to DMSO control wells) were correlated with values obtained from the biochemical AlphaScreen and the cellular AlphaLISA assays

Finally, as part of our ongoing lead optimization, we next selected 18 random inhibitors, which displayed a 3-log range in potency in cellular AlphaLISA assays, and evaluated their activity in OV90 cells using the in situ cell extraction assay (Fig. 6). Upon measuring their potency against endogenous TRIM24 in OV90 cells (Fig. 6b), we next correlated the EC_50_ values with those obtained from the cellular TRIM24/histone H3 binding assay (AlphaLISA) as well as the biochemical AlphaScreen peptide-displacement assay (Fig. 6c). Importantly, we observed correlation coefficients (*R*^2^ > 0.6) for these complementary assays, further supporting the use of the in situ cell extraction protocol for endogenous drug-target engagement assessment.

### In situ cell extraction as a platform to dissect the contribution of individual domains to chromatin binding

**Fig. 7:**
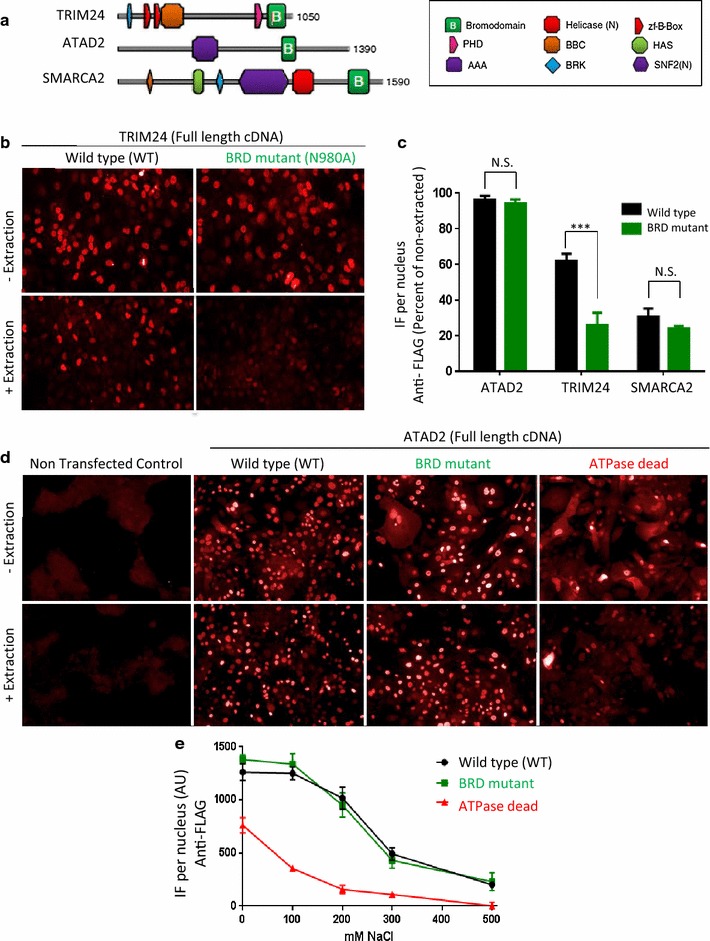
In situ cell extraction applied to genetic studies of chromatin binding for candidate drug targets TRIM24, SMARCA2 and ATAD2. **a** Schematic representation of proteins highlighting the multi-domain nature of select bromodomain-containing genes. **b** A functional TRIM24 bromdomain is required for chromatin binding. IF images of HeLa cells expressing FLAG-tagged wild-type (WT) or bromodomain-binding-deficient mutant (BRD mut) forms of full-length TRIM24 under in situ cell extraction (+) and non-extraction (−) conditions. **c** Quantification of the indicated FLAG-tagged full-length proteins (ATAD2, TRIM24 and SMARCA2) in the nuclei of transfected HeLa cells after in situ cell extraction (CSK buffer with 100 mM NaCl) for wild-type (*black*) and bromodomain mutant (*green*) proteins (n = 3; similar results observed across different cell lines including HeLa, hMECs and A549; data not shown). **d** Representative IF images of hMECs expressing FLAG-tagged full-length (WT) and mutant ATAD2 constructs (BRD mut and ATPase dead) followed by IF detection of the ATAD2 transgene (i.e., anti-FLAG antibody). **e** Quantification of the nuclear signals for ATAD2 wild-type (WT), BRD mutant and ATPase dead proteins after in situ cell extraction using increasing NaCl stringency. Changing the stringency of the in situ cell extraction (0–500 mM NaCl) does not change the relative rank order of their chromatin-binding affinities

The bromodomain drug discovery field is complicated by the fact that many of these proteins contain multiple conserved domains, involved in chromatin binding and protein–protein interactions, and by the fact these proteins are frequently part of large protein complexes (Fig. 7a). Therefore, we next wanted to explore whether the in situ cell extraction technique could be used as a general strategy to decipher the contribution of individual bromodomains to chromatin biology. To this end, we focused on TRIM24, SMARCA2 and ATAD2 as they represent prominent candidates for anti-cancer drug discovery [13, 31–34]. Focusing on full-length proteins, we expressed FLAG-tagged wild-type (WT) and bromodomain-binding-deficient mutant (BRD mut) constructs in cells and subjected these to the in situ cell extraction protocol followed by IF quantification (Fig. 7b, c).

Consistent with pharmacological TRIM24 inhibitor studies, the bromodomain mutant form of full-length TRIM24, displayed a drastically reduced affinity for chromatin, compared to the WT TRIM24 counterpart (Fig. 7c; black and green bars, respectively). As such, this mutational study confirmed the essential role of an intact bromodomain in the ability of TRIM24 to bind chromatin in cells. For SMARCA2, about 30 % of the ectopically expressed full-length protein was present (i.e., chromatin bound) following the in situ cell extraction step compared to the non-extracted IF signal, but the bromodomain mutation (BRD mut) did not significantly alter this level. The observation that the SMARCA2 bromodomain is not required for chromatin binding is also consistent with pharmacological inhibitor studies using the SMARCA2 chemical probe PFI-3 [35]. Likewise, for ATAD2, no significant difference in chromatin binding was observed between the WT and bromodomain mutant constructs (Fig. 7c). However, when we overexpressed various mutant forms of ATAD2, we noted that the nuclear localization of ATAD2 was significantly altered in cells expressing an ATPase dead construct (compared to WT and BRD mut constructs), as exemplified by studies in human mammary epithelial cells (hMECs) (Fig. 7d). In these cells, chromatin binding of ATAD2 was assessed under increasingly stringent in situ cell extraction conditions ranging from 0 to 500 mM NaCl (Fig. 7e). Contrary to the ATAD2 WT and BRD mut proteins, which displayed similar affinities to each other for chromatin structures, the ATPase dead protein resided primarily in the nuclear soluble fraction, removed by the in situ cell extraction procedure, revealing a dependency on functional ATPase activity for chromatin binding and hence subnuclear distribution. In conclusion, the in situ cell extraction assay is a powerful cell-based methodology to evaluate not only bromodomain inhibition, but also to dissect the role/contribution of different domains in chromatin binding and to provide critical insights into both drug-target discovery and basic science.

## Conclusions

Multiple biochemical assays have been utilized to support the discovery of small-molecule inhibitors of epigenetic enzymes, but the development of cellular binding assays for protein–protein interactions involving the so-called ‘reader’ domains has lagged behind. Cellular assays are critically important for the identification and optimization of potent inhibitors suitable for in vivo pharmacology studies and subsequent clinical development. While protein stabilization and fluorescence resonance energy transfer (FRET) assays have been developed for BRD2/4 to detect interaction with small-molecule inhibitors in engineered cells [7, 8, 36], no universal cell-based drug-target engagement assays amenable to 384-well HTS format have been reported to date for bromodomains.

In this manuscript, we describe two new strategies to measure the cellular interaction between bromodomain and chromatin; namely (1) proximity-based binding (AlphaLISA), and (2) in situ cell extraction and fluorescence-based, high-content imaging. Our detailed protocols (Additional file 1: Tables S1, S2 and S3) add to the repertoire of epigenetic cellular assays, paving the way for the development of novel chemical probes. Because protein–protein interactions regulate numerous cellular functions that go awry in cancer, the disruption of interactions related to chromatin modifiers and remodeling complexes has been a desirable goal for drug discovery in cancer as well as other pathological conditions. In principle, our novel assays can be applied to any drug target that specifically binds to histones or chromatin/nuclear structures.

We have applied the in situ cell extraction platform to diverse proteins and have noticed that many bromodomains, in isolation from their flanking protein modules, are often cytoplasmic or, if nuclear, non-chromatin bound in the absence of SAHA treatment. Similar observations have been reported for a FRET bromodomain probe [36] and more recently in studies using FRAP analysis for a dozen bromodomains [9]. Although we find that short-term HDAC inhibitor treatment is a useful ‘trick’ for developing robust bromodomain-binding assays in transfected cell lines, it is clear that SAHA treatment, in addition to increasing the H3K23Ac mark, also hyper-acetylates other histone- and non-histone-associated proteins [37, 38]. Consequently, transfected and ectopically expressed bromodomain constructs may be recruited to non-physiological binding sites. Supporting this notion is our general observation that for full-length bromodomain-containing genes, including TRIM24, ATAD2 and SMARCA2/4, stable cell lines tend to display low levels of overexpression (one- to threefold compared to endogenous) and their binding to chromatin is not significantly influenced by SAHA treatment compared to the expression of truncated, isolated bromodomain constructs. As shown for TRIM24-PB, the isolated bromodomain fragment expresses well in stable cell lines, potentially because it engages protein complexes within cells differently as compared to its full-length counterpart (Fig. 3). Nevertheless, for cell-based drug-target engagement assays, aiming to identify and guide the selection and design of potent, cell-permeable chemical probes, the issue of binding aberrant lysine recognition motifs is less of a concern. For lead identification and optimization processes, sensitivity, robustness, ease of automation and low cost are critical in a HTS setting (384-well plate of higher), as lead molecules are typically evaluated downstream in complementary cellular and functional assays looking at disease-relevant phenotypes.

From a drug discovery and proximal PD biomarker perspective, the highest value of the in situ cell extraction assay, when using antibody-based IF, resides in the opportunity to monitor displacement of endogenous full-length proteins from physiological sites in the nucleus, thereby correlating drug-target engagement with anti-cancer phenotypes (e.g., apoptosis, differentiation and migration) in any target cell of interest. Specifically, when applied to our TRIM24 drug discovery project, the ability to monitor displacement of endogenous full-length TRIM24 from chromatin in adherent cell line enables pivotal PK/PD/efficacy studies to ensure that the compound phenotype (i.e., anti-viability) is not due to off-target effects, but rather linked to on-target TRIM24 activity.

Although the ‘low resolution’ histone- and chromatin-binding assays (i.e., AlphaLisa and in situ cell extraction) developed here are well suited for compound screening, the ability to map the genome-wide locations of proteins and drug targets at ‘high resolution’ is critical for our understanding of normal and disease biology. While not amenable to high-throughput screening, global analysis methods such as ChIP-seq and Chem-seq are important approaches to define how target proteins and small-molecule inhibitors may perturb specific gene expression program [39, 40]. In fact, models of the transcriptional regulatory circuitry that controls normal and disease states in cells have emerged from genome-wide Chip-Seq and Chem-Seq data [35, 39–43] and this global analysis represents important follow-up assays for small-molecule drug discovery programs targeting transcription factors, chromatin regulators and other epigenetic proteins.

The truncated TRIM24 PHD-bromodomain sequence is predicted to encode two naturally occurring NLS motifs within its Carboxy-terminal tail and, interestingly, appending NLS sequences to other GFP-tagged proteins have been used to streamline FRAP assay development for a dozen bromodomains [9]. We also found that addition of a NLS sequence to an isolated GFP-tagged SMARCA2 bromodomain construct is necessary for developing in situ cell extraction assay for SMARCA2. Using this assay, we recently characterized a novel bromodomain inhibitor, PFI-3, and reported that while PFI-3 can displace the isolated GFP-tagged SMARCA2 bromodomain from chromatin in engineered cells, it fails to displace the endogenous full-length SMARCA2 protein from chromatin [35]. This has significant implications for small-molecule drug discovery as SMARCA2 knock-down causes cancer-specific, phenotypic lethality in SMARAC4-null lung cancers [31–33], but bromodomain inhibition does not phenocopy the effects of RNAi-mediated target depletion [35]. Hence, we propose that the in situ cell extraction assay can be used to prioritize and functionally assess the importance of individual bromodomains for chromatin complex formation.

Finally, given the absence of pharmacological inhibitors for many bromodomain targets, site-directed mutagenesis can also be used to rapidly dissect the contribution of individual domains to chromatin binding, as illustrated here for ATAD2, where the ATPase domain, but not the bromodomain, is driving chromatin association. Such results can directly inform on drug discovery efforts and, in the case of ATAD2, we predict that such endeavors should be directed towards inhibiting the more challenging ATPase catalytic domain to mimic the reported RNAi phenotype [34]. As such, we believe the in situ cell extraction platform has significant implications for dissecting the role of individual conserved domains, present in a diverse family of emerging epigenetic and chromatin-modifying proteins beyond the bromodomain family (e.g., Tudor domains [41, 42]). Altogether, the quantitative, cell-based approaches for measuring bromodomain–histone and chromatin interactions, presented herein, combined with both genetic and emerging pharmacological tools, are poised to help prioritizing protein families for drug discovery and contribute to the delivery of novel epigenetic therapies for human diseases.

## Methods

### Plasmids and chemicals

Bromodomain mutations were made in human TRIM24 (N980A) using site-directed mutagenesis (QuickChange, Agilent Technologies) with related binding-deficient mutations in ATAD2 (GeneCopoeia #GC-Z4424) and SMARCA2 (GeneCopoeia #GC-Z4424). The ATAD2 ATPase dead mutations (K473T and E532Q) were as described elsewhere [43]. All cDNAs, subcloned into lentiviral vectors (pLEX304; AddGene #25890), were sequence verified and virus generation, infection and generation of stable cell lines were conducted following standard procedures. The FLAG-TRIM24-PB construct (residues 810–1050) was generated by PCR amplification and cloned in-frame with the V5-tag present in pLEX304. Unless otherwise noted, all chemicals, including Trichostatin A (TSA) and Suberoylanilide Hydroxamic Acid (SAHA) were from Sigma-Aldrich. Synthesis of the TRIM24 bromodomain inhibitor, IACS-6558, with the following IUPAC name *N*-{1,3-dimethyl-6-[3-(2-methylpropoxy)phenoxy]-2-oxo-2,3-dihydro-1H-1,3-benzodiazol-5-yl}-1,2-dimethyl-1H-imidazole-4-sulfonamide, and IACS-9571, is described elsewhere [14].

### Biolayer interferometry and AlphaScreen binding assays

The His-tagged TRIM24 PHD-bromodomain was expressed and purified as previously described [13]. The H3K23Ac peptide (AnaSpec, CA, USA) used for biolayer interferometry (Red-384, ForteBio, CA, USA) and AlphaScreen (Perkin Elmer) was: [ARTKQTARKSTGGKAPRKQLATK(ac)AARKSAPATG-YK(Biotin)]. For biolayer interferometry, Streptavidin ‘dip-and-read’ biosensors (ForteBio, #18-5019) were loaded with the biotinylated-H3K23Ac peptide in Buffer A (20 mM Hepes, pH 7.5, 150 mM NaCl, 1 mM TCEP, 0.005 % Tween-20). The biosensors were then washed in Buffer A and transferred into 96-well plates (Greiner #655209) containing a 7-point, 1.5-fold serial dilution of the TRIM24 protein (starting from a 67 nM solution in Buffer A). The *K*_*D*_ for the TRIM24/H3K23Ac interaction was calculated from the *k*_off_/*k*_on_ values obtained by kinetic fitting of the association and dissociation curves using the OctetRed software. AlphaScreen assays were conducted in Buffer B (50 mM Hepes pH 7.4, 100 mM NaCL, 0.1 % BSA, 0.05 % Chaps) using an automated 384-well protocol (Additional file 1: Table S1). In brief, from a (1.5×) master mixture of TRIM24 protein (7.5 nM) and H3K23Ac (22.5 nM), 8 µL was dispensed into 384-well OptiPlates and incubated with 4 µL of test compound (or DMSO control) for 1 h at room temperature (RT). Streptavidin-donor beads and nickel chelate (Ni–NTA) acceptor beads (8 µL; 25 μg/mL) were incubated for 2 h at RT prior to measuring the AlphaScreen signal (Envision plate reader, PerkinElmer). All dose–response graphs (four-parameter logistic curve) and IC_50_ values were obtained using GraphPad Prism 6 software.

### Immunoprecipitation

HeLa cells (confluent 10 cm dish) were washed with PBS and harvested in 1 mL of Buffer B (10 mM TRIS pH 7.5, 10 mM KCl, 1 mM CaCl2, 0.1 % Triton X-100). Lysates were sonicated, treated with 15 units micrococcal nuclease (Worthington Biochemical Corporation #LS004798) at RT for 10 min. Supernatant was collected following centrifugation at 14,000*g* for 10 min. Immunoprecipitation of FLAG-tagged TRIM24 was performed using FLAG M2 antibody conjugated magnetic beads (Sigma #M8823). Beads (25 µL) were incubated overnight at 4 °C with 2 mg of the whole cell extract. Beads were collected and washed three times with Buffer C (50 mM TRIS pH 7.5, 1 mM EDTA, 50 mM NaCl, 0.5 % NP40) and two times with Buffer D (20 mM TRIS pH 8, 1 mM EDTA, 150 mM NaCl, 1 % NP40, 1 % Triton X-100, 0.5 % sodium deoxycholate). The washed beads were boiled in 2× protein loading dye and subjected to SDS-PAGE immunoblot analysis using the following antibodies: FLAG-HRP (Sigma #A8592), H3K23ac (Active Motif #39131), H3K4me2 (Active Motif #39141), H3 (Abcam #1791) and Lamin B (Santa Cruz #6217). For immunoprecipitation of endogenous full-length TRIM24, cell extracts were incubated overnight at 4 °C with 4 μg rabbit IgG or TRIM24 antibody (Proteintech #14208-1-AP). Protein A Sepharose beads (30 μL; GE Healthcare) equilibrated in buffer C were incubated with the extracts for 1 h at 4 °C to precipitate immune complexes. Beads were washed three times with buffer C and two times with buffer D, boiled in 2X protein loading dye and subjected to SDS-PAGE immunoblot analysis.

### AlphaLISA

Hela TRIM24-PB cells were maintained in DMEM medium supplemented with 10 % FBS and 5 µg/mL of Blasticidin. Cells were seeded (10,000/well, 40 µL) to 384-well white culture plates (PerkinElmer, #6007680) using a Multidrop 384 reagent dispenser (Thermo Scientific) and incubated overnight at 37 °C and 5 % CO_2_. On Day 2, the cells were co-treated with SAHA (10 µM) and test compound and incubated at 37 °C and 5 % CO_2_ for 2 h. The plates were washed twice with PBS (60 µL at RT) using a Biomek FX liquid handler (Beckman Coulter). Lysis buffer (10 µL, Invitrogen #FNN0011), supplemented with protease and phosphatase inhibitors (ThermoScientific #1861282), was added to the plate using a Multidrop and the plates were sealed and spun down, followed by shaking (15 min, 700 rpm at RT) using an Eppendorf tabletop mixer. Next, histone extraction buffer (10 µL, PerkinElmer #AL009F2) diluted tenfold in water was added to all wells, followed by mixing to ensure complete extraction. Anti-histone H3 antibody (5 µL; 3 nM) diluted in PBS containing 1 % BSA was added to each well before the plates were sealed, spun down and incubated for 30 min at RT. Anti-FLAG acceptor (PerkinElmer #AL112 M) and Streptavidin-donor (PerkinElmer, #6760002) beads were diluted and mixed together in the Manufacture-provided Detection Buffer to generate a 40 µg/mL master suspension. From here, 10 µL of acceptor and donor bead mixture was added to each well, before the plate was sealed and incubated in the dark for 2 h at RT. Plates were read on Envision plate reader (PerkinElmer) using AlphaScreen protocol and all IC_50_ values (four-parameter logistic curve) were obtained using GraphPad Prism 6 software. See Additional file 1: Table S2.

### In situ cell extraction

OV90 or HeLa cells (20,000 cells/100 µL) were seeded into 96-well plates (Corning Costar #3603) using RPMI or DMEM medium, respectively, supplemented with 10 % FBS and grown overnight. Test agents were serially diluted in DMSO (with or without SAHA) to generate 10X master compound plates. From here, 11 µL was transferred into each well containing 100 µL of growth media followed by shaking on a rotation table (15 min, 300 rpm at RT) and incubation at 37 °C for 2 h. Plates were washed once with PBS (150 µL/well) and then with cold, freshly prepared Cytoskeleton (CSK) Buffer (10 mM PIPES, 300 mM Sucrose, 100 mM NaCl, 3 mM MgCl_2_; pH = 6.8). Immediately thereafter, soluble, non-chromatin-bound proteins were extracted by addition of cold CSK Buffer supplemented with 0.5 % Triton X-100 (150 µL per well) for 10 min at 4 °C followed by 4 % paraformaldehyde fixation (10 min at RT) (Additional file 1: Table S3).

### Proximity ligation assay (PLA)

Hela cells expressing FLAG-TRIM24-PHD-Bromo-V5 (TRIM24-PB) (20 K/100 µL) were seeded into 96-well plates (Corning Costar #CLS3603) in DMEM media supplemented with 10 % FBS and grown overnight. After treatment with SAHA (5 µM, 2 h), plates were washed with PBS (150ul per well) and fixed with 4 % paraformaldehyde (10 min at RT). PLA was performed using Duolink^®^ In situ Red Starter Kit Mouse/Goat (Sigma-Aldrich, DUO92103), according to manufacturer’s recommendation. Primary antibody pair was: Goat anti-V5 (1:200 dilution; Bethyl #A190-119A) and Mouse anti-histone H3 (1:4000 dilution; Active Motif, #39763). Following PLA, secondary antibody Alexa Fluor^®^594 Donkey Anti-Goat (Invitrogen #A-11056) and Alexa Fluor^®^488 Donkey Anti-Mouse (Invitrogen #A-21202) were used to measure the total levels of V5 and histone H3. Using the High-Content Screening System Operetta with 40× magnification, cell images from 25 fields in each well were taken in 4 channels (Alexa488, Alexa594, Alexa647 and Hoechst). The image analysis was performed in Harmony software by selecting ‘nuclei’ and ‘cytoplasm’ in Hoechst channel and ‘dots within nucleus’ in Alexa647 channel. Gating for single cells of flat morphology was done based on nuclei area, roundness and intensity of Hoechst in the nucleus and in the cytoplasm. For each gated cell, we calculated the mean signal intensity for histone H3 (Alexa488 in the nucleus minus Alexa488 in the cytoplasm); TRIM24 (Alexa594 in the nucleus minus the Alexa546 intensity in the cytoplasm); and the mean number for the PLA signal (number of spots in nucleus). For each fluorophore, values were averaged across all the gated cells (Additional file 1: Figure S2c).

### Immunofluorescence and high-content image analysis

Standard IF procedures were used. Briefly, cells were permeabilized (0.5 % Triton X-100 in PBS) for 15 min and incubated with blocking solution (DAKO #X0909) for 30 min. The following primary antibodies were used: goat anti-V5 (Bethyl #A190-119A) 1:200; mouse anti-FLAG (Sigma #F1804) 1:200; rabbit anti-BRD4 (Abcam #ab128874) 1:200; rabbit anti-TRIM24 (Proteintech #14208-1-AP) 1:400; rabbit anti-K3K23Ac (Cell Signaling #8848) 1:200, and mouse anti-histone H3 (Active Motif #39763) 1:4000. Alexa Fluor-conjugated secondary antibodies were from Invitrogen and Hoechst 33342 (Invitrogen, #H3570) was used for nuclei counterstaining (10 µg/ml). For each Alexa fluorochrome, images (4–10 fields per well; 20X magnifications) were acquired using the Operetta High-Content Screening System (PerkinElmer). Image analysis was performed in Harmony software (PerkinElmer) by selecting nuclei and cytoplasm in Hoechst channel. Gating for single cells of flat morphology was done based on nuclei area, roundness and intensity of Hoechst in the nucleus and in the cytoplasm. In every gated cell, the mean signal intensity value for each fluorochrome was calculated separately in nucleus and cytoplasm. The specific nuclear signal was estimated subtracting mean cytoplasmic value from nuclear; and average value was calculated using at least 1000 cells in each well.

### Statistical analysis

For the homogeneous, bead-based AlphaScreen and AlphaLISA binding studies dose–response curves are displayed as Percent of Control (POC) calculated according to the following formula: POC = ([sample signal-average background signal]/[average maximum signal − average background signal]) × 100. All EC_50_ values were calculated based on these POC values using four-parametric nonlinear regression (i.e., curve fitting) software (either Genedata Screener™ software version 11 or Graphpad Prism 6). For the cell-based AlphaLISA assay, the POC values were calculated using the following wells and process: (1) a “blank subtraction” was performed by subtracting the mean signal of the blank control wells (which contained media but lacked cells) from all of the measured values; (2) these corrected values were then used in the POC formula stated above. The “average background signal” corresponds to wells that contain cells but lacked SAHA treatment while the average “maximum signal” corresponds to cells treated with SAHA. For the biochemical AlphaScreen assay, the background signal corresponds to wells lacking TRIM24 protein and H3 peptide and the maximum signal is derived from DMSO control wells with both the TRIM24 protein and peptide. For the in situ cell extraction assay, single cell analysis was performed using a grid of 9 non-overlapping rectangular fields projected in silico onto the image from each well. From these representative fields, at least 1000 cells were analyzed per well and each experimental condition was represented by at least three independent wells. Average and standard Deviation (SD) values were calculated using the Harmony software (PerkinElmer). For presentation purposes, the data from Harmony were also exported to GraphPad Prism and analyzed using the build-in global nonlinear regression tool (four-parametric curve fitting) to visualize dose–response curves and EC_50_ values.

## Additional file

**Additional file 1.** Six supplementary figures and three supplementary tables.

## Abbreviations

BET: bromodomain and extraterminal domain family
IACS: Institute for Applied Cancer Science
SGC: structural Genomics Consortium
TRIM24: tripartite motif containing 24
BRD4: Bromodomain-containing protein 4
ATAD2: ATPase family, AAA domain containing 2
SMARCA2: SWI/SNF-related, matrix-associated, actin-dependent regulator of chromatin, subfamily A, member 2
HATs: histone acetyltransferases
HDACs: histone deacetylases
PTMs: post-translational modifications
FRAP: fluorescence recovery after photobleaching
GFP: green fluorescent protein
ATPase: adenosinetriphosphatase
EZH2: enhancer of zeste homolog 2
AlphaLISA/AlphaScreen: Amplified luminescent proximity homogeneous assay
HTS: high throughput screening
PHD: plant homeodomain
WT: wild-type
FL-TRIM24: full-length TRIM24
PB-TRIM24: PHD and bromodomain of TRIM24
SAHA: suberoylanilide hydroxamic acid
PLA: proximity ligation assay
S/B: signal to background
