# Supplementary material for: Development of novel cellular histone-binding and chromatin-displacement assays for bromodomain drug discovery
Source: Epigenetics Chromatin. 2015 Sep 21;8:37. doi: 10.1186/s13072-015-0026-4 (PMC4578755; doi:10.1186/s13072-015-0026-4)
Supplement: Supplementary file 1 — Additional file 1. Six supplementary figures and three supplementary tables. [file 13072_2015_26_MOESM1_ESM.pdf]

Additional File 1 (Supplementary Figures and Tables)

Development of Novel Cellular Histone-binding and Chromatin-displacement Assays for Bromodomain Drug Discovery

Yanai Zhan[1*], Maria Kost-Alimova[1*], Xi Shi[1], Elisabetta Leo[1], Jennifer P. Bardenhagen[1], Hannah E. Shepard[1], Srikanth Appikonda[2], Bhavatarini Vangamudi[1], Shuping Zhao[1], Trang N. Tieu[1], Shiming Jiang[2], Timothy P. Heffernan[1], Joseph R. Marszalek[1], Carlo Toniatti[1], Giulio Draetta[1], Jessica Tyler[2], Michelle Barton[2], Philip Jones[1], Wylie S. Palmer[1], Mary K. Geck Do[1#], Jannik N. Andersen[1#].

[1]Institute for Applied Cancer Science, The University of Texas MD Anderson Cancer Center, Houston, TX, 77230, USA.

[2]Department of Molecular Carcinogenesis and Epigenetics, The University of Texas MD Anderson Cancer Center, Houston, TX, 77230, USA.

[*]Equal contributors

[#]**Address Correspondence:**

Mary K Geck Do: mkgeck@mdanderson.org

Jannik N. Andersen: jandersen@xtuit.com

Current address: XTuit Pharmaceuticals, 700 Main Street, Cambridge, MA 02139. USA.

Running Title: Cellular Assays for Bromodomain Drug Discovery.

Supplementary Figure Legends

Figure S1. Small-molecule HTS screen using a typical bromodomain-peptide displacement assay. Percent AlphaScreen signal relative to DMSO control (POC) for a focused library of lysine mimetic compounds screened against the bromodomain of ATAD2 using the active-site peptide ligand (H4K5Ac). Select hits from the primary screen were subsequently validated using label-free orthogonal binding assays (Octet) and through ATAD2 x-ray co-crystallization studies highlighting the utility of this biochemical assay platform (Poncet-Montange, et al., 2015).

Figure S2. Proximity Ligation Assay (PLA) showing co-localization of V5-tagged TRIM24 with endogenous histone H3 in cells. (A) Schematic illustration of the PLA principle. (B) Three parameters were experimentally measured in untreated (-SAHA) and treated (+SAHA) (2 µM, 2h) Hela TRIM24-PB cells: (i) V5-tagged TRIM24 (pink) using IF with goat anti-V5 and secondary anti-goat Alexa594 antibodies; (ii) histone H3 (green) using mouse anti-histone H3 and secondary anti-mouse Alexa488 antibodies; (iii) the interaction between TRIM24 and histone H3 (blue dots) by PLA using the above V5 and anti-histone H3 antibody pair for proximity ligation. Images were captured using Operetta and analyzed using Harmony software, calculating the mean intensity of Alexa488 and Alexa594 and an average number of PLA spots per nucleus. (C) Quantification of the PLA signal as number of spots per nucleus showing a ~4-fold increase in the detected co-localization events upon SAHA treatment. The total protein levels of TRIM24 in the nuclei (i.e. Mean IF intensity for anti-V5 per nucleus) remain unchanged in response to SAHA treatment.

Figure S3. JQ1 rapidly displaces BRD4 from chromatin as measured by in situ cell extraction. Hela cells expressing FLAG-tagged bromodomain of BRD4 were treated by JQ1 (1 µM) during different time intervals (10 time points). After *in situ* cell extraction, the chromatin bound BRD4 was measured using anti-FLAG antibody, and the mean IF intensity per nucleus was plotted. The effect of treatment (decrease in BRD4 bromodomain binding to chromatin) was seen already after 5 min.

Figure S4. Proof-of-concept for miniaturization of the in situ cell extraction assay. (A) The *in situ* cell extraction can be easily transferred into 384-well format. The table insert shows the

number of fields and the number of cells in 96- and 384-well format that is enough for analysis. (B) BRD4 inhibitor studies (384-well plate format) show that the dose-response curves are comparable with the 96-well format (Fig. 4) in terms of rank ordering of compounds producing a Z' value of 0.74.

Figure S5. HTS-friendly GFP-version of the in situ cell extraction assay. (A) *In situ* cell extraction using TRIM24 PB-GFP fusion protein significantly simplifies the chromatin binding assay. (B) The cells were treated with TRIM24i in 96-well plate using concentration range from 20 nM to 100 µM. After 1h incubation, extraction was performed with Hoechst 33342 added into the extraction buffer (10 min), then cells were fixed and scanned by Operetta. EC_{50} curve was calculated by Harmony software during the scan. (C) Plate-view after Operetta analysis shows separately GFP (green), Hoechst (red) and superimposed images. (D) The magnified field shows the cells treated or untreated with TRIM24 inhibitor. The same field of view is shown before and after extraction. Note full displacement of TRIM24-GFP from chromatin under TRIM24i treatment. This displacement occurs very rapid, during 1 min (data not shown).

Figure S6. *In situ* cell extraction on HeLa TRIM24-GFP allows monitoring TRIM24 soluble protein extraction in live time. (A) Images showing the kinetics of the *in situ* cell extraction step using the TRIM24-GFP fusion protein expressed in HeLa cells. Extraction buffer was added to the wells and each field of view was monitored every 3 minutes during 15 minutes. (B) The EC_{50} values obtained for the TRIM24 inhibitor (TRIM24i or IACS-6558) are robust to variation in the duration of the *in situ* cell extraction step providing a significant window for experimental design (4-13 min).

Supplementary Fig. S1 (Zhan *et al*.)

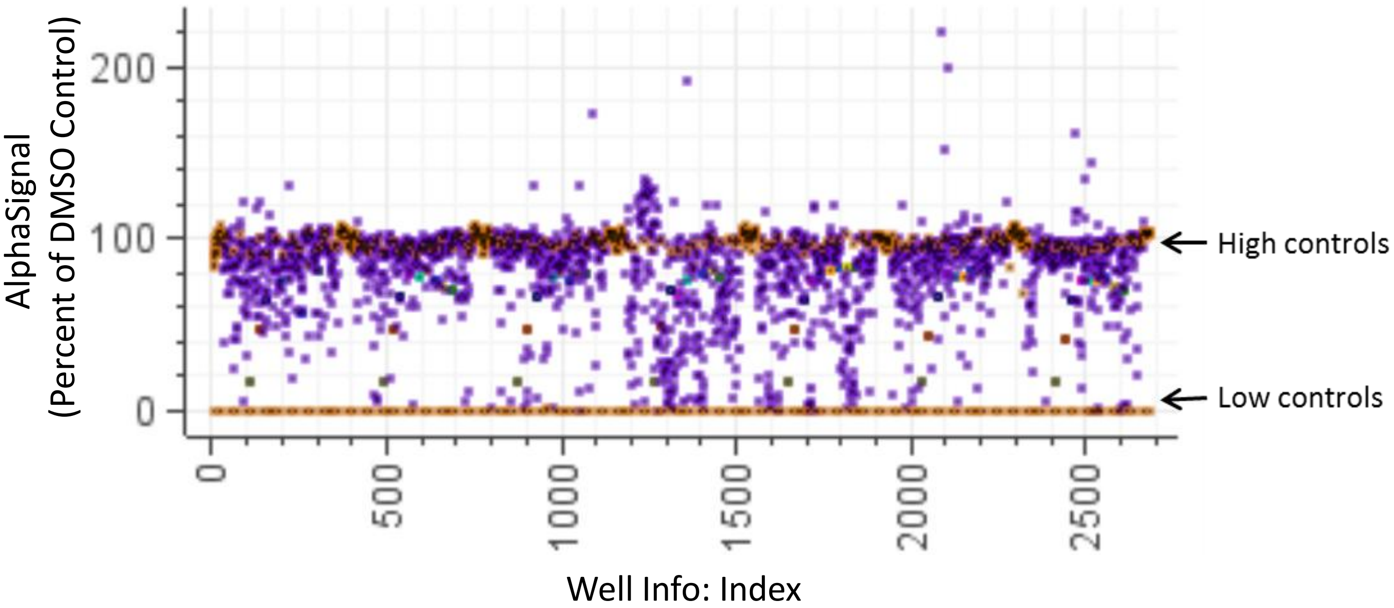

Supplementary Fig. S2 (Zhan *et al.*)

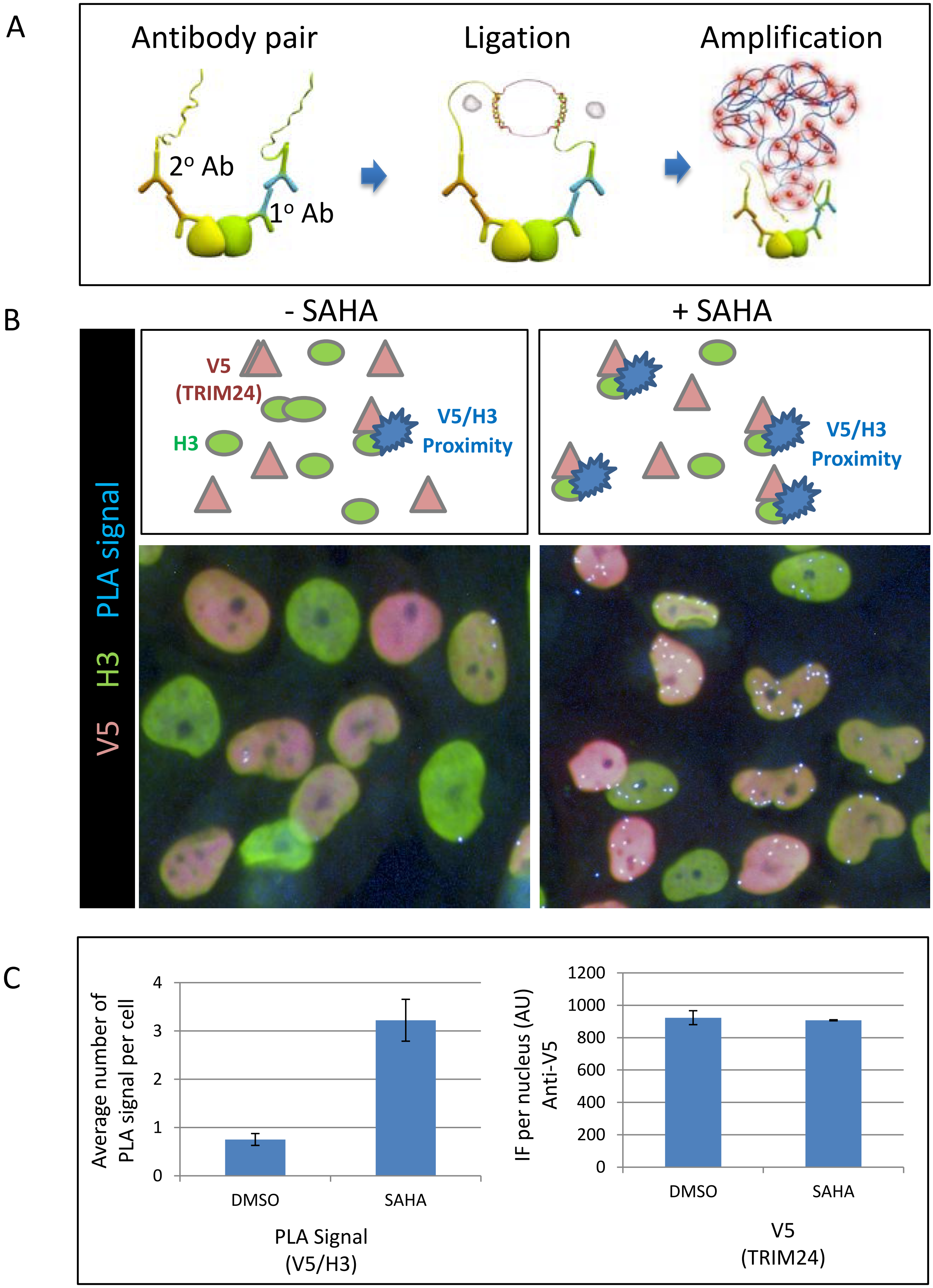

Supplementary Fig. S3 (Zhan *et al.*)

A

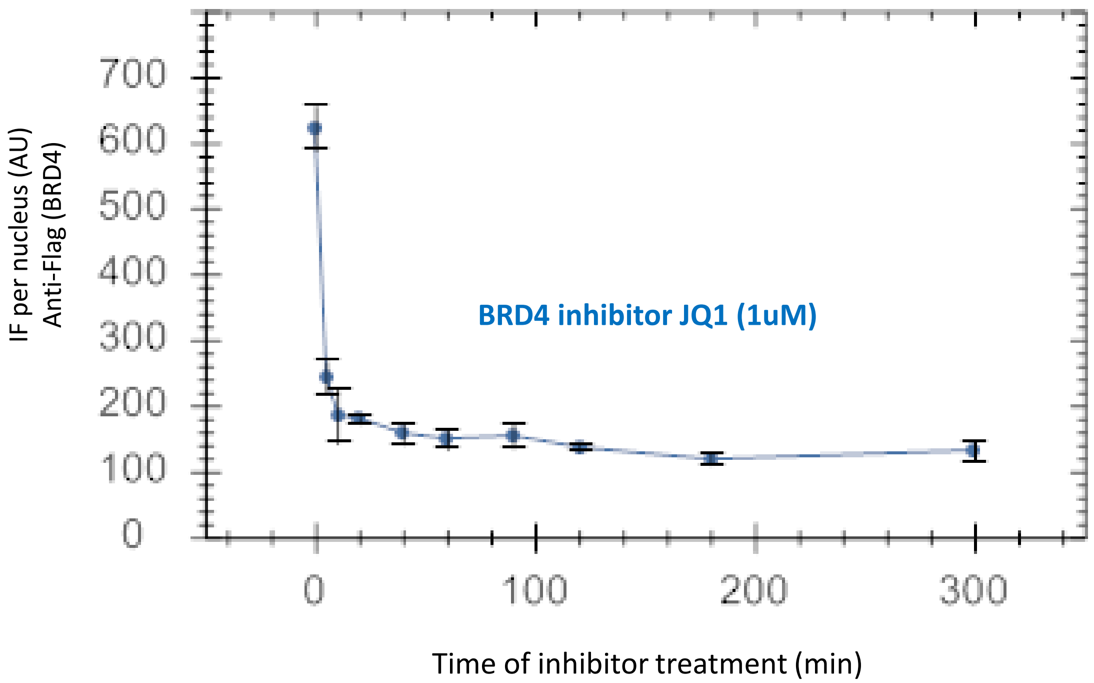

Supplementary Fig. S4 (Zhan *et al.*)

A

Parameter	96-well	384-well
No of cells seeded (Day 0)	20,000	5,000
No of cells per field used for analysis	100-300	100-300
Total no of cells needed for quantification	>1000	>1000
Total no of fields needed for quantification	4-10	4-10
Total no of fields per well	60	15

B

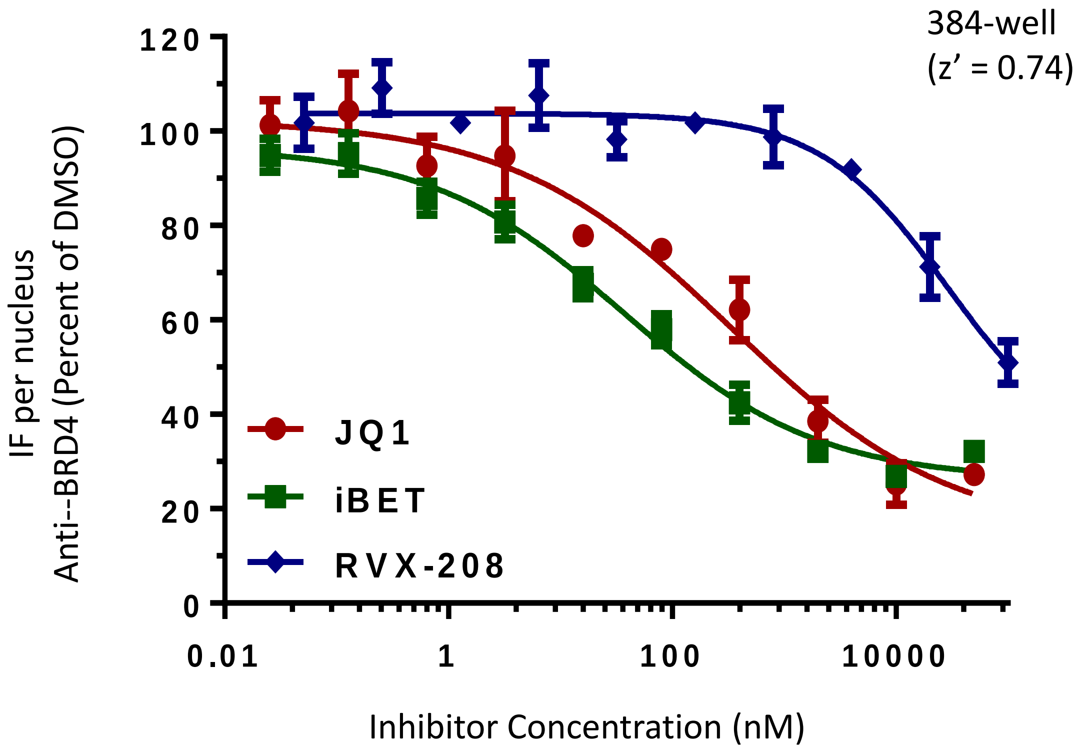

Supplementary Fig. S5 (Zhan *et al.*)

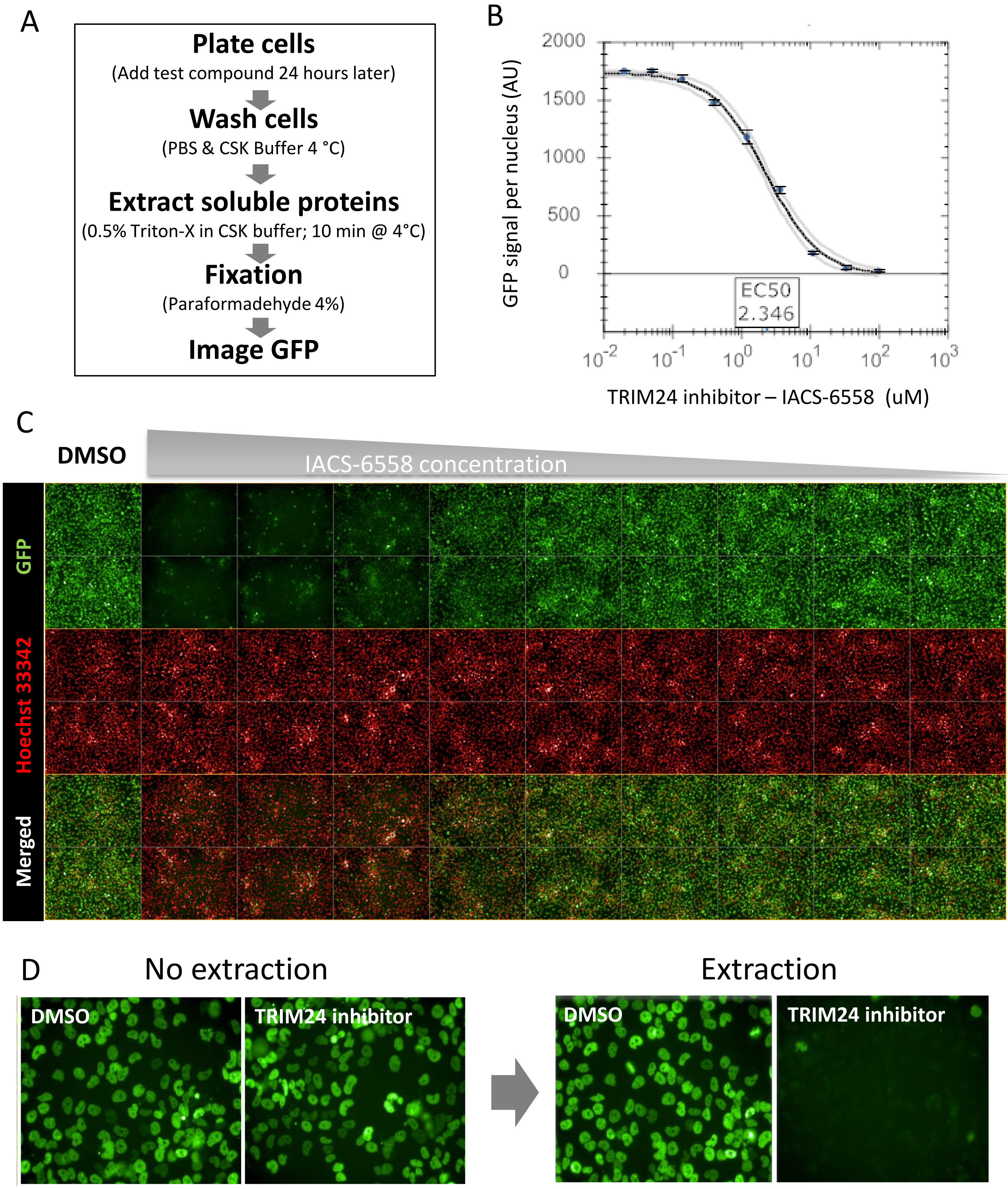

Supplementary Fig. S6 (Zhan *et al.*)

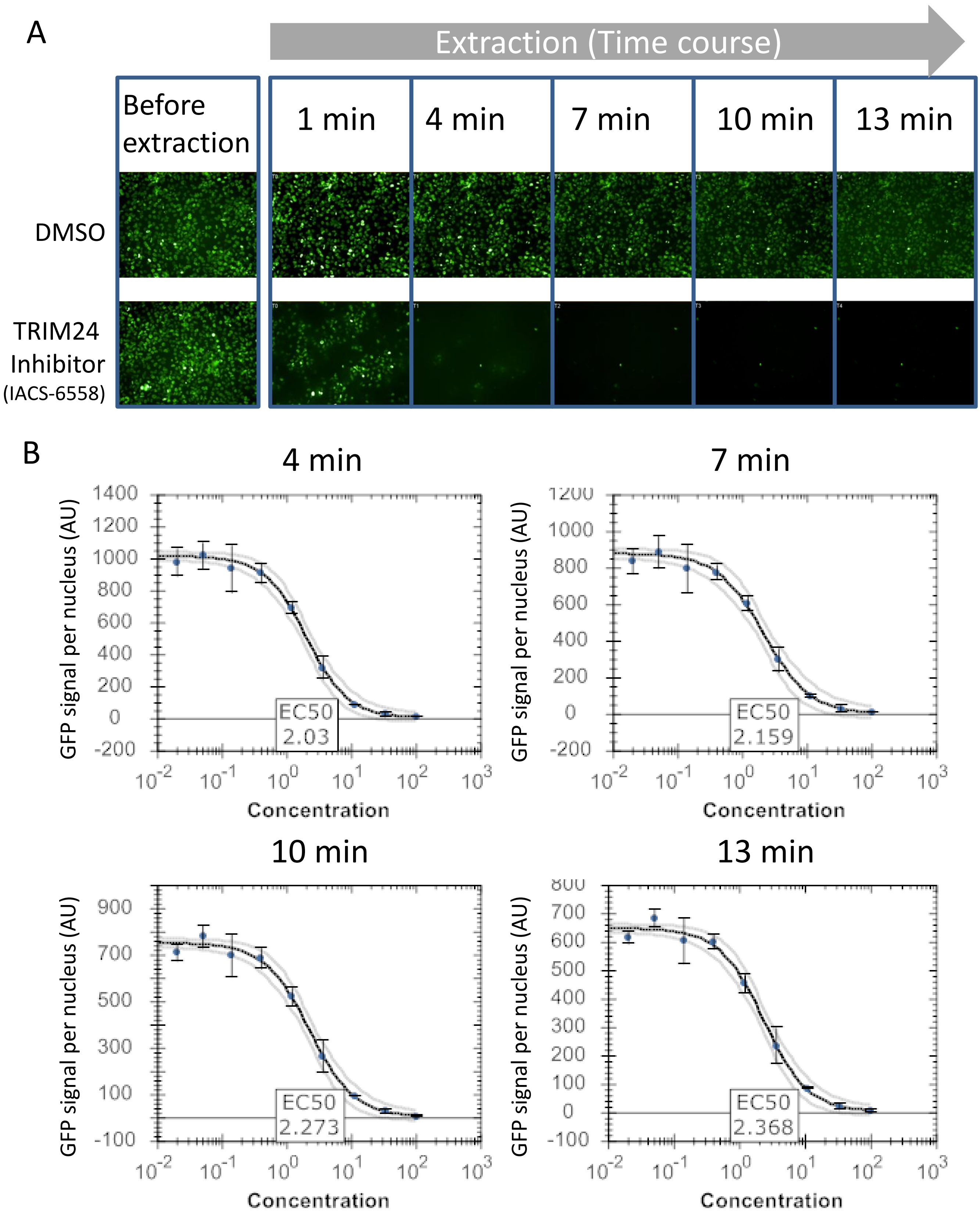

Table S1. AlphaScreen protocol for TRIM24/H3K23Ac peptide binding assay (384-well format)

Step	Parameter	Value	Description
**1**	Prepare Master Compound Plate (i.e. test substances/inhibitors)	30 µL	200X final concentration in assay Three-fold serial dilution (100% DMSO)
**2**	Prepare dilution of Master Compound Plate	101.5 µL	1.5 µL from Step 1 is added into 100 µL of assay buffer in polypropylene plates and mixed
**3**	Dispense His-TRIM24-PB protein and biotinylated histone H3 peptide	8 µL	1.5X solution of TRIM24 protein (7.5 nM) and H3K23Ac (22.5 nM) peptide (OptiPlate)
**4**	Dispense compounds into TRIM24/peptide solution	4 µl	Compounds from Step 2 dispensed into OptiPlate
**5**	Incubation time	1 hr	RT, sealed (Perkin Elmer Top Seal)
**6**	Dispense AlphaScreen detection beads	8 µl	2.5X master mix of Streptavidin donor and Nickel Chelate Ni-NTA acceptor beads
**7**	Incubation time	2 hrs	RT, sealed, in dark
**8**	AlphaScreen assay read out	615 nm	Envision plate reader, AlphaScreen mode

Reagents (Catalog numbers): Perkin Elmer: 384-well OptiPlate (#6007299); AlphaScreen NKL-ChLT kit (#6760619M); Top Seal (#6005250); Sigma: BSA (#A2153) and Chaps (#C5070).

Step Notes:

1. Three-fold serial dilution in DMSO (100 %) in a polypropylene plate (10 points total)
3. Final assay concentrations are 5 nM His-TRIM24 and 15 nM Biotinylated H3K23Ac peptide, prepared in Assay Buffer B (50 mM Hepes pH 7.4, 100 mM NaCl 0.1 % BSA, 0.05 % Chaps).
4. Final concentration of DMSO in the assay is 0.5 % (v/v).
6. Final concentration of AlphaSreen detection beads (acceptor & donor master mix) is 10 ug/mL (prepared in the dark using 1 x AlphaScreen Detection Buffer).

Table S2. AlphaLISA protocol for cellular TRIM24/histone H3 binding assay (384-well protocol)

Step	Parameter	Value	Description
**1**	Seed cells (Hela TRIM24-PB) in 384-well pLates	40 µL	10,000 cells/well; 40 µL of DMEM supplemented with 10 % FBS (Perkin Elmer white CulturePlate)
**2**	Incubation time	Overnight	37°C and 5% CO_2
**3**	SAHA treatment	5 µL	From 1% DMSO Stock (90 µM in DMEM/10% FBS)
**4**	Prepare Master Compound Plate (100X)	30 µL	Three-fold serial dilution in DMSO in a polypropylene plate, 10 points total
**5**	Prepare dilution of Master Compound Plate (10X)	33.3 µL	3.3 µL from step 4 is added into 30 µL of media in a new polypropylene plate
**6**	Dispense compounds	5 µL	Dispense compounds from Step 5 into CulturPlates
**7**	Incubation time	2 hr	37°C and 5% CO_2
**8**	Wash plate	60 µL	RT PBS (2 times), leave 5ul of PBS in the well
**9**	Dispense Lysis Buffer	10 µL	Lysis buffer containing freshly added protease and phosphatase inhibitor cocktail
**10**	Incubation time	15 min	RT, sealed, shake at 700rpm
**11**	Dispense diluted Histone Extraction Buffer	10 µL	Histone Extraction Buffer diluted 1:10 using water
**12**	Dispense anti-Histone H3 antibody	5 µL	6X anti-histone H3 antibody diluted in PBS containing 1% *(w/v)* BSA
**13**	Incubation time	30 min	RT, sealed, shake at 500rpm
**14**	Dispense AlphaLisa detection beads	10 µL	4X mix of Streptavidin donor and anti-Flag acceptor beads in 1X Cell Histone Detection Buffer
**15**	Incubation time	2 hr	RT, sealed, in dark
**16**	Assay read out	615 nm	Envision plate reader, AlphaScreen mode

Reagents (Catalog Numbers). Invitrogen: Cell Lysis Buffer (#FNN0011); Perkin Elmer: 384-well Culture Plates (#6007680), Cell Histone Extraction Buffer (#AL009F2), Cell Histone Detection Buffer (#AL009F3), Biotinylated anti-H3 antibody (#AL118M), Anti-Flag Acceptor Beads (#AL112M), Anti-Strepavidin Donor Beads (#6760002).

Step Notes:

3. Final assay concentration of SAHA is 10 µM, with 0.1% (v/v) DMSO
4. Master Compound plates are 100X of final assay concentration
6. Upon completion of this step, the DMSO concentration in the assay is 1.1% (v/v)
9. Cell Lysis Buffer is prepared by adding ThermoScientific cocktail inhibitor (1:100) to Invitrogen Cell Extraction Buffer (FNN0011)
10. Centrifuge (1000 rpm, 1 min.) before and after shaking.
11. Mix thoroughly to ensure complete extraction.
12. Final assay concentration of anti-histone H3 antibody is 0.5 nM.
14. Final concentration of beads in the assay is 10 µg/mL (acceptor and donor master mix; 1:1 ration). Beads are prepared in the dark using 1X AlphaLISA Cell-Histone Detection Buffer.
16. Plates are sealed and kept dark.

Supplemental Table S3 (Zhan *et al.*)

Table S3. *In Situ* Cell Extraction Procedure (IF)

Step	Parameter	Solution	Volume	Time	Temp (°C)
**1**	Seed cells (20,000 cells per well)	DMEM supplemented with 10 % FBS (in 96-well plates)	100 µL	o/n	37°C, 5%CO_2
**2**	SAHA treatment (optional)	SAHA (50 µM) in DMEM with 10% FBS	12 µL	2h	37°C, 5%CO_2
**3**	Prepare Master Compound Plate (100X)	100X final concentration in assay	30 µL		
**4**	Prepare dilution of Master Compound Plate (10X)	3.3 µL from step 4 is added into 30 µL of media in a new polypropylene plate	33.3 µL		
**5**	Compound treatment	Dispense compounds from Step 5 into Culture Plates	12 µL	2h	37°C and 5% CO_2
**6**	Washing	PBS, CSK	150 µL	Short	4°C
**7**	Extraction	0.5% TritonX100 in CSK Buffer	150 µL	10 min	4°C
**8**	Fixation	4% PFA in PBS	100 µL	10 min	RT
**9**	Washing	PBS	150 µL	5 min	RT
**10**	Protein block	Protein blocking solution	70 µL	30 min	4°C
**11**	Primary antibody	Diluted in antibody diluent	40 µL	o/n	4°C
**12**	Washing	0.05% Tween20 in PBS	150 µL	2x5min	RT
**13**	Secondary antibody	Diluted in antibody diluent	40 µL	60 min	37°C
**14**	Washing	0.05% Tween20 in PBS	150 µL	2x5min	RT
**15**	Washing	PBS	150 µL	Short	RT
**16**	Scanning and image analysis	*See below			

(Skip steps 9-14 for GFP-fusion proteins)

Reagents (Catalog Numbers). Cytoskeleton Buffer (10mM PIPES, 300mM Sucrose, 100mM NaCl, 3mM MgCl2; pH 6.8); 96-well plates (Corning Costar #3603); Paraformaldehyde, 16% Solution (Electron Microscopy Sciences #3294843); Protein blocking (DAKO #X0909); Antibody diluent (DAKO # S080983-2)

Primary antibodies and dilutions used: goat anti-V5 (Bethyl #A190-119A) 1:200; mouse anti-FLAG (Sigma #F1804) 1:200; rabbit anti-BRD4 (Abcam #ab128874) 1:200; rabbit anti-TRIM24 (Proteintech #14208-1-AP) 1:400; rabbit anti-K3K23Ac (Cell Signalling #8848) 1:200, and mouse anti-histone H3 (Active Motif #39763) 1:4000.

Image acquisition: Images from nine fields (20X) in each well were taken Using Operetta High Content Screening system (PerkinElmer) .

Image analysis: The analysis was performed in Harmony (PerkinElmer) software by selecting nuclei and cytoplasm in Hoechst 33342 channel . Gating for single cells of flat morphology was done based on nuclei area, roundness and intensity of Hoechst in nucleus and in cytoplasm. For each channel "mean nuclear signal" was calculated as "mean intensity in nucleus" –" mean intensity in cytoplasm" and averaged per nucleus .
